# Establishing a new rat model to investigate pathophysiology and bone healing in posttraumatic lymphedema

**DOI:** 10.1371/journal.pone.0332067

**Published:** 2025-09-16

**Authors:** Felix Reinkemeier, Christoph Wallner, Marius Drysch, Sonja Verena Schmidt, Flemming Puscz, Yonca Steubing, Ali Bakri, Carsten Theiss, Marcus Lehnhardt, Bjoörn Behr, Johannes Maximilian Wagner

**Affiliations:** 1 Department of Plastic Surgery and Hand Surgery, BG-University Hospital Bergmannsheil, Bochum, Germany; 2 Department of Cytology, Institute for Anatomy, Ruhr-University Bochum, Bochum, Germany; 3 Department of Plastic, Reconstructive and Aesthetic Surgery, EVK Essen, Essen, Germany; Universidade dos Açores Departamento de Biologia: Universidade dos Acores Departamento de Biologia, PORTUGAL

## Abstract

**Introduction:**

Posttraumatic lymphedema is a common complication after open and closed fractures with soft tissue trauma. Even though there is quite certain consensus about the basic mechanisms of lymphedema formation, the pathophysiology on a cellular and molecular base is largely unknown. Furthermore, there is currently no data on the interaction of lymphedema and bone regeneration. Subsequently, the aim of this study was to establish an animal model specific to posttraumatic lymphedema, which can be used to conduct future investigations into the pathophysiology and bone regeneration in lymphedema.

**Methods:**

The test animals (rats)In wild-type Fisher 344 rats were divided into three groups: group one had an isolated bone defect, group two had an isolated soft tissue defect, and group three had a combination of bone and soft tissue trauma.

**Results:**

Using volumetric and circumferential measurements, a significant increase in circumference and volume in the sense of lymphedema could be detected, particularly 3–4 weeks after trauma in the groups with soft tissue trauma, whereas the creation of a bony defect did not appear to have a significant influence on the swelling. Microscopic images of the lymphatic drainage pathways verified the lymphatic drainage disorder weeks after soft tissue trauma.

**Conclusion:**

Consequently, the established model can be used to investigate the exact pathophysiology of post-traumatic lymphedema. Furthermore, it seems to be suitable as a model for investigating bone regeneration in manifest lymphedema.

## 1. Introduction

Lymphedema is a chronic inflammatory condition of the interstitial tissue caused by impairment of the lymphatic drainage system. It is classified into primary and secondary forms based on etiology [1,2]. Primary lymphedema results from congenital hypoplasia or dysfunction of the lymphatic vessels, whereas the more common secondary lymphedema arises from acquired damage to the lymphatic system. Major contributing factors include malignancies and associated lymph node dissection, radiation therapy, infections, trauma, and obesity [3–5].

Accurate epidemiological data on secondary lymphedema remain limited; however, its incidence in industrialized countries is estimated at 0.13–2%. According to the German Society for Lymphology, approximately 80,000 individuals in Germany are affected [6]. Considering early epidemiological studies on posttraumatic lymphedema – reporting that up to 50% of patients with open lower extremity fractures develop chronic lymphedema symptoms [7] – the prevalence following trauma, and thus the overall prevalence of secondary lymphedema, may be significantly underestimated.

Diagnosis is typically based on clinical findings (e.g., volumetry, limb circumference) and a thorough medical history, including predisposing factors such as malignancy or trauma. The hallmark of manifest lymphedema is marked swelling, characterized by a significant increase in volume and circumference of the affected extremity. Although most cases of secondary lymphedema are unilateral, bilateral presentation is also possible. Additional features include skin hardening and progressive tissue fibrosis.

From a pathophysiological perspective, lymphedema results from an insufficient lymphatic drainage system, which disrupts the physiological balance between fluid filtered through the blood vessel walls and the lymphatic system’s transport capacity. As a result, interstitial fluid volume increases and its composition changes [1].

Lymphedema is recognized as a progressive disease, driven by two key mechanisms. First, excessive fluid accumulation in the interstitium compresses surrounding venous vessels, increasing hydrostatic pressure. This promotes further fluid extravasation from the venous system into the tissue, thereby perpetuating a vicious cycle of pressure-induced leakage. Second, lymph fluid is rich in proteins, leading to elevated colloid osmotic pressure in the interstitial space, which further facilitates fluid movement from the capillaries into the tissue [3,8].

Over time, impaired tissue perfusion results in irreversible fibrotic remodeling, hypertrophy of adipose tissue, and atrophy of lymphatic smooth muscle cells [3,4,8,9]. In advanced stages, trophic skin changes may occur, including epidermal hyperplasia with hyperkeratosis, mild papillomatosis, and verrucous protrusions. These pathological changes form the basis for secondary complications such as recurrent wound healing disturbances and ulceration [10].

In summary, while there is broad consensus regarding the basic pathophysiological mechanisms of lymphedema development, knowledge about the underlying cellular and molecular processes remains fragmented and largely speculative.

Most experimental studies to date have focused on general molecular mechanisms underlying lymphedema; however, a specific investigation of posttraumatic lymphedema has not yet been conducted. To develop effective pharmacological treatments, further basic research is needed to identify the key cell types and cytokines involved in the pathogenesis of posttraumatic lymphedema. In particular, a better understanding of the temporal dynamics of these processes is essential to optimize the timing and efficacy of future therapeutic and prophylactic interventions.

In recent years, several animal models of lymphedema have been developed across various scientific disciplines. These models typically induce isolated lymphatic injury through targeted lymphadenectomy or radiotherapy. While such methods closely mimic clinical scenarios like oncologic resection, lymph node dissection, or radiation-induced lymphedema, they do not adequately reflect the complex injury patterns observed in posttraumatic lymphedema, which frequently involves extensive soft tissue damage and associated fractures.

As previously discussed, most studies on lymphedema prevalence and pathophysiology have focused on malignancy-associated forms. Given that the mechanisms underlying posttraumatic lymphedema remain poorly understood, the development of a clinically relevant animal model is of particular importance. Rodent models have gained increasing relevance in lymphedema research, especially over the past four to five years. In small animals, both superficial and deep lymphatic vessels are located within the dermis [11,12], meaning that a skin incision alone can cause significant lymphatic disruption. By combining such injury with an established femoral bone defect model, this approach enables the simultaneous investigation of bone regeneration under conditions of lymphatic system impairment.

There is therefore a clear need for an animal model that replicates lymphatic damage in a trauma-based context. The model presented in this study not only provides initial insights into the development of posttraumatic lymphedema, but also allows for the examination of its interaction with bone healing.

The initial hypothesis was that microscopic disruption of lymphatic vessels, combined with soft tissue trauma, would induce lymphedema in the lower extremity. This would be reflected by a progressive increase in limb circumference and volume over time compared to the contralateral side. It was further assumed that an isolated bone defect would not significantly affect these parameters.

Despite the growing interest in lymphedema research, current animal models predominantly focus on lymphatic injury in oncologic contexts and do not adequately capture the complex interplay between mechanical trauma, soft tissue damage, and lymphatic disruption. Notably, there is a lack of standardized small-animal models that simulate posttraumatic lymphedema as it occurs following high-energy injuries with concomitant fractures. This gap limits our ability to study the pathophysiological cascade unique to trauma-induced lymphedema and its potential impact on tissue healing, particularly in the musculoskeletal system. The present model addresses this need by providing a reproducible and clinically relevant platform to investigate both the development of lymphedema and its influence on bone regeneration under posttraumatic conditions.

## 2. Materials and methods

Wild-type Fischer 344 rats aged 12–16 weeks were used for the experiments, housed in groups of 4–6 animals. A total of 30 animals (15 male, 15 female) were randomly assigned to three groups (n = 10 per group). Group 1 (control) received an isolated bone defect in the right tibia. Group 2 underwent soft tissue injury including transection of the lymphatic vessels to induce lymphedema. In Group 3, both interventions were combined.

The contralateral (left) limb served as an internal control to minimize variability due to individual differences in body size, particularly in circumference and volume measurements.

### 2.1. Reduction of animal suffering

All procedures were performed under sterile conditions in a dedicated animal surgery room on a sterile-draped operating table. Preoperative analgesia was administered 45 minutes before surgery using buprenorphine (0.05 mg/kg i.p.), followed by repeated doses every 4 hours during the day and via drinking water (0.009 mg/ml) overnight for 24 hours postoperatively. Tramadol was used for subsequent analgesia.

Anesthesia was induced in a chamber with 2.5 vol% isoflurane in oxygen (10 ml/min). After loss of postural reflexes, animals were placed on a heated surgical table and maintained under anesthesia via face mask with isoflurane (2.0–2.5 vol%), oxygen (2 ml/min), and nitrous oxide (0.5 ml/min).

Animal breeding and housing were performed and monitored by trained personnel in accordance with institutional standards. All experimental procedures were conducted by qualified staff and designed to ensure that animals were only exposed to the minimum necessary burden. Appropriate anesthesia and analgesia protocols were employed to minimize pain and distress. At the end of the observation period, animals were euthanized humanely with minimal stress. Anesthesia, analgesia, and euthanasia protocols followed the recommendations of GV-SOLAS regarding experimental procedures, administration routes and volumes, and humane endpoints.

All experiments were performed in adherence to the National Institute of Health guidelines for the use of experimental animals and after approval by the German legislation. The protocol was approved by the Landesamt für Natur, Umwelt und Verbraucherschutz (NRW, Germany; permit-number: 81–02.04.2022.A292).

### 2.2. Operations of the control and experimental groups

**Fig 1:**
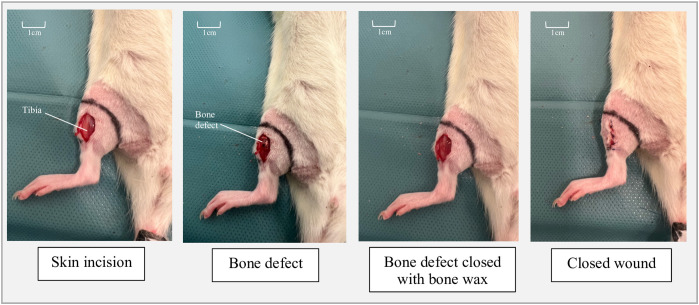
Surgical steps in the intervention group 1 and 3.

A 1–2 cm longitudinal skin incision was made over the right medial tibia in wild-type Fischer 344 rats. The posterior tibial muscle was bluntly separated, and the anterior border of the tibia was exposed. A unicortical hole (1 mm in diameter) was drilled into the anterior tibial cortex using a pin drill (Ultimate NSK 450). The surgical site was irrigated with sterile saline, the muscle layers were repositioned, and the skin was sutured and disinfected (Fig 1).

**Fig 2:**
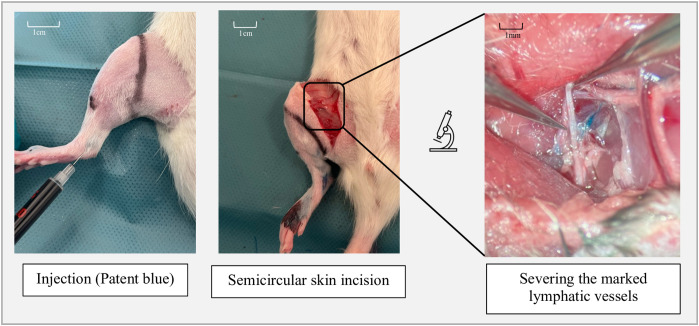
Surgical steps in the intervention group 2 and 3.

Next, approximately 0.05 ml of Patent Blue was injected intradermally around the right ankle joint to visualize the lymphatic vessels. To simulate soft tissue trauma, a semicircular skin incision (~2.5 cm) was made on the medial thigh, extending through the cutaneous soft tissue layers down to the underlying musculature. This step alone already disrupts superficial lymphatic vessels (Fig 2).

The remaining procedure was performed under an operating microscope. The lymphatic vessels running within the subcutaneous tissue of the inner thigh were carefully exposed and then transected to further compromise lymphatic drainage and induce lymphedema. Finally, the surgical site was irrigated and the skin closed. The contralateral (left) hind limb remained untouched and served as a control.

The first group (control group) included animals that received only the bone defect; thus, only the first surgical step was performed. Visualization of lymphatic structures was not required in this group. In the second group (lymph group), only soft tissue injury and transection of the lymphatic vessels were performed. In the third group (bone + lymph group), both procedures were carried out to induce lymphedema in combination with a bone defect. All surgical interventions were performed exclusively on the right hind limb.

### 2.3. Measurements

Circumference measurements were taken preoperatively and then weekly for four weeks at five anatomical landmarks: metatarsus, ankle, lower leg, popliteal region, and thigh. Measurements were performed bilaterally to allow side-by-side comparison of the right (operated) and left (control) hind limbs. In addition, volumetric analysis of the hind limbs was conducted.

Diameter was measured using a digital caliper with 0.01 mm precision (TOP Craft DMV-SL05), and circumference was assessed using a calibrated tape measure. For volume determination, the hind limbs were immersed in water-filled tubes; the displaced fluid was collected and weighed with a precision scale (accuracy 0.01 g, G&G Pocket Scale LS2000H). Diameter and volume were recorded weekly over the same four-week period.

At the end of the observation period, animals were re-anesthetized, and all measurements were repeated. Finally, Patent Blue was reinjected, and lymphatic drainage was visualized under a surgical microscope.

All measurements were performed under general anesthesia.

### 2.4. Statistics

Statistical analysis was performed using data from all 10 animals per group. Results are presented as mean ± standard deviation (SD). Normal distribution was assessed using the Shapiro–Wilk test, and homogeneity of variances was evaluated with the F-test. For comparisons between two groups, Student’s t-test was applied. For multiple group comparisons, one-way ANOVA followed by Tukey’s post hoc test was used. A p-value of < 0.05 was considered statistically significant.

## 3. Results

**Fig 3:**
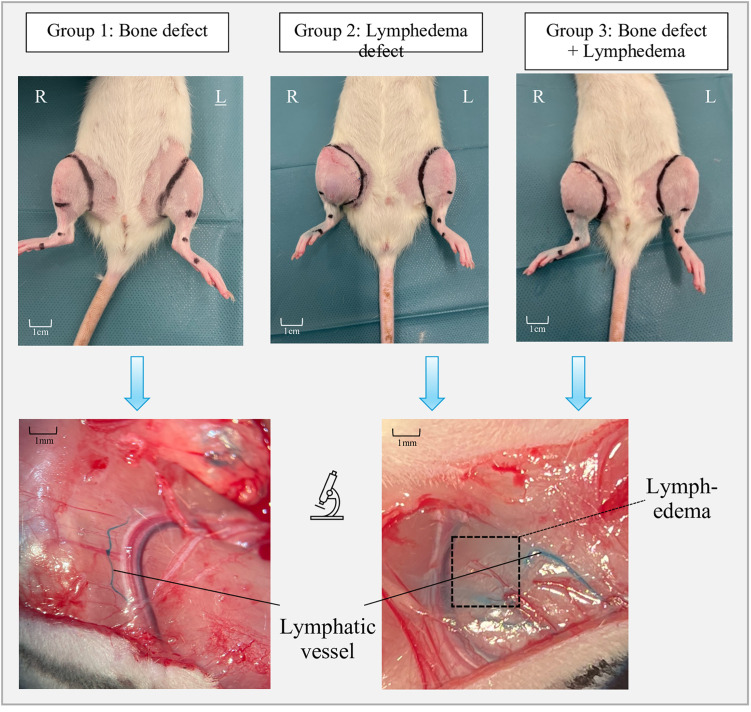
Macroscopic and microscopic representation of the legs in side and group comparison.

Four weeks postoperatively, animals in the lymph and bone + lymph groups showed a significant increase in circumference and volume of the right hind limb compared to the contralateral side (Fig 3). In contrast, no macroscopic swelling was observed in the control group. Following intradermal injection of Patent Blue, intact lymphatic drainage was visualized in the control group. In the lymph and bone + lymph groups, however, no such drainage was detectable, consistent with the development of lymphedema (Fig 3).

**Fig 4:**
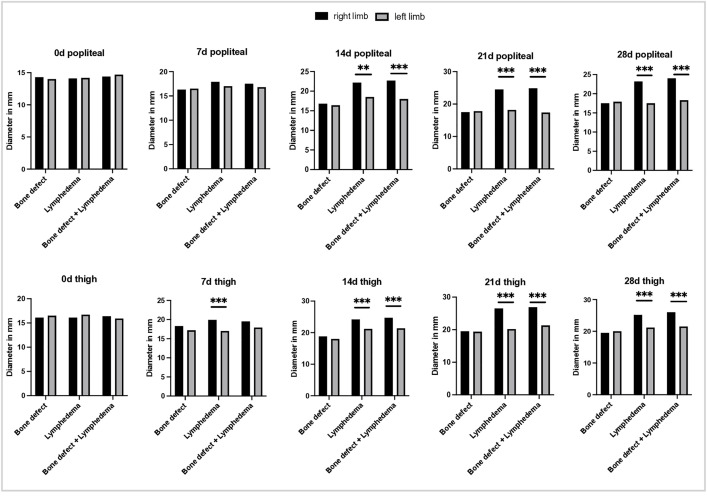
Comparison of leg diameter before surgery (day 0), as well as 7,14,21 and 28 days postoperatively. Above: diameter of the legs at the level of the knee joint. Below: diameter at the level of the thigh. The black bars represent the measurements of the right hind leg, the gray values of the left hind leg.

**Fig 5:**
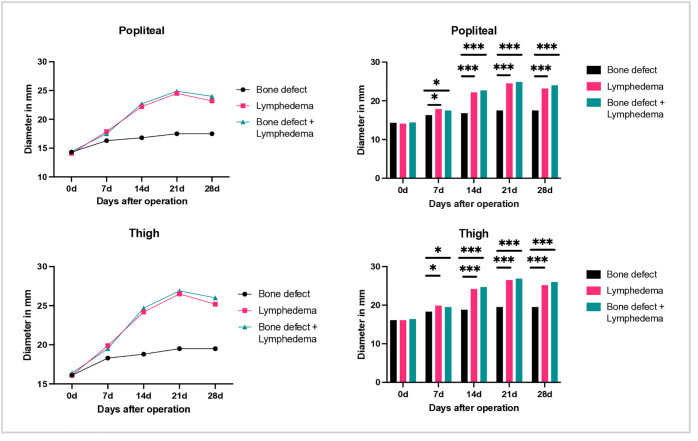
Representation of right leg diameter before surgery (day 0), as well as 7,14,21 and 28 days postoperatively. Above: diameter of the legs at the level of the knee joint. Below: diameter at the level of the thigh. Left: visualization of the diameter over time. Right: comparison between the groups.

No significant differences in diameter were observed at the metatarsus, ankle joint, or lower leg levels in any group during the four-week postoperative period. However, by day 7, animals in the lymph and bone + lymph groups showed increased diameters at the popliteal region and thigh, compared both to the contralateral (left) side (Fig 4) and to the control group with isolated bone defect (Fig 5). The maximum difference in leg diameter at the popliteal and thigh levels was observed at week 3. Interestingly, a slight decrease in diameter at both sites was noted between days 21 and 28.

**Fig 6:**
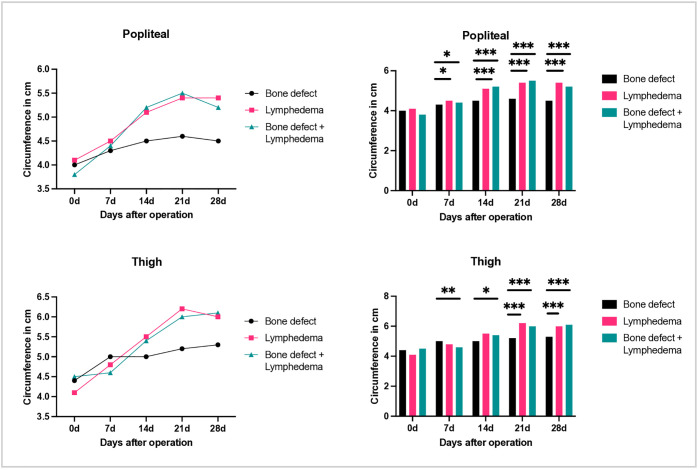
Representation of right leg circumference before surgery (day 0), as well as 7,14,21 and 28 days postoperatively. Above: circumference of the legs at the level of the knee joint. Below: circumference at the level of the thigh. Left: visualization of the diameter over time. Right: comparison between the groups.

As with diameter measurements, no significant differences in circumference were observed below the knee joint. In contrast, animals in the lymph and bone + lymph groups showed significantly increased circumference at the popliteal region and thigh in side-by-side comparison with the contralateral limb (Fig 6). Additionally, both soft tissue injury groups differed significantly from the control group with isolated bone defect. No statistically significant differences in circumference were observed between the lymph and bone + lymph groups.

**Fig 7:**
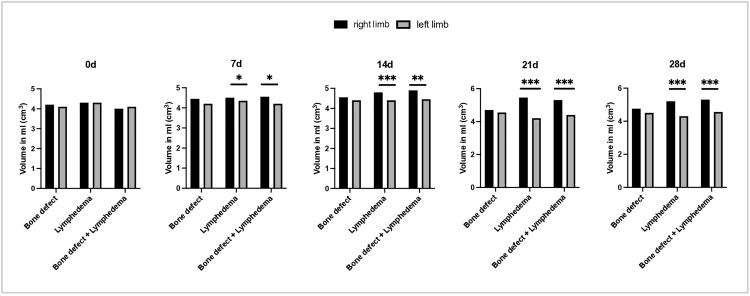
Comparison of leg volume before surgery (day 0), as well as 7,14,21 and 28 days postoperatively. Above: volume of the legs at the level of the knee joint. Below: volume at the level of the thigh. The black bars represent the measurements of the right hind leg, the gray values of the left hind leg.

**Fig 8:**
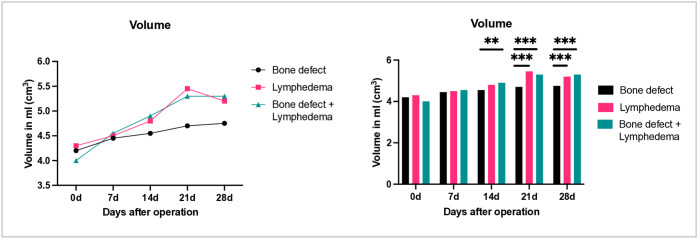
Representation of right leg volume before surgery (day 0), as well as 7,14,21 and 28 days postoperatively. Left: visualization of the volume over time. Right: comparison between the groups.

Finally, volumetric measurements were performed on both hind limbs. In the lymph and bone + lymph groups, a significant increase in volume of the operated limb was observed from day seven onwards in side-by-side comparison with the contralateral side (Fig 7). Maximum volume was recorded at approximately three weeks postoperatively. In intragroup comparison, the volume of limbs subjected to soft tissue injury was significantly higher than that of the contralateral limb (Fig 8). In contrast, no significant volume differences were observed in the control group with isolated bone defect over the 28-day observation period.

## 4. Discussion

The aim of this experimental study was to establish an animal model of posttraumatic lymphedema that allows for deeper insights into its pathophysiology and the development of novel therapeutic and prophylactic strategies. In addition, the model was designed to investigate bone regeneration and its interaction with manifest lymphedema.

In line with these objectives, the study demonstrated that impairment of lymphatic drainage in combination with soft tissue trauma leads to a measurable increase in limb circumference and volume in rats. Based on the clinical definition of lymphedema as swelling caused by impaired lymphatic outflow, the observed changes are consistent with posttraumatic lymphedema. Furthermore, it was shown that the isolated tibial bone defect had no significant influence on these parameters—e.g., due to hematoma formation.

Although various models for studying lymphedema have been described, none specifically address posttraumatic lymphedema. As early as 2005, lymphatic regeneration following amputation and subsequent replantation was investigated in a canine model [13]. Histopathological and lymphographic analyses revealed initial signs of lymphatic regeneration after approximately 9–10 days.

In recent years, various mouse models have been described, particularly from Asia and Scandinavia. In one approach, lymphedema was induced by surgical lymphatic obstruction combined with two fractions of 10 Gy irradiation [14]. Another model employed a single 10 Gy irradiation followed by ligation of three lymphatic vessels and excision of regional lymph nodes [15]. In a further study, superficial inguinal, popliteal, and deep inguinal lymph nodes were surgically removed, and the femoral lymphatic vessel was transected [16]. In the same model, improved lymphatic drainage was reported following hyaluronidase injection.

A similar technique was described by Iwasaki et al., who induced hindlimb lymphedema by resecting the inguinal and popliteal lymph nodes along with adjacent fat pads, followed by the implantation of a silicone splint in the groin region [17]. In the context of oncological research on head and neck tumors, a mouse model was developed in which lymphedema was induced by combining radiation therapy with cervical lymph node dissection [18]. Additionally, in the field of reconstructive lymphatic surgery, a rat model was established based on the isolated removal of the popliteal lymph node [19]. After the development of unilateral lymphedema, vascularized lymph node transfer (VLNT) was simulated by transplanting the contralateral popliteal lymph node.

In recent years, several new animal models have been developed to advance lymphedema research, addressing both methodological and therapeutic aspects. Morita et al. introduced a simple, reproducible, and long-lasting rat model that is particularly suitable for standardized investigations [20]. Building on such foundational models, therapeutic interventions have increasingly been integrated. For example, Nguyen et al. combined a surgically induced rat model with the implantation of a collagen-based medical device for targeted treatment of lymphedema [21]. At the same time, the clinical relevance and methodological quality of existing models have come under closer scrutiny. Podder et al. evaluated the translational potential of animal models for lymphatic dysfunction [22], while Bucan et al. conducted a systematic review and quality assessment of surgically induced hindlimb lymphedema models in mice [23]. In parallel, new imaging approaches have emerged: Nikolaev et al. applied optical coherence tomography to objectively assess lymphatic status in a rat limb model [24]. Finally, Hoshino et al. demonstrated the therapeutic potential of the prolyl hydroxylase inhibitor roxadustat, showing enhanced lymphatic regeneration in a mouse hindlimb lymphedema model [25].

While these models have contributed significantly to the understanding of lymphatic disruption and chronic fluid accumulation, they primarily simulate oncologic scenarios and do not adequately reflect the complex injury patterns seen in trauma patients. Notably, the above-mentioned models often involve localized and controlled lymphatic injury without accompanying damage to adjacent soft tissue or skeletal structures. In contrast, the present study introduces a rat model that incorporates both a standardized soft tissue injury and a segmental bone defect, better simulating the multifactorial pathophysiology of posttraumatic lymphedema. By inducing diffuse lymphatic disruption within a clinically relevant trauma context, this model offers a more representative platform to study the interplay between lymphatic damage and bone healing processes.

In our model, the results obtained from all three measurement methods were largely consistent. Significant increases in circumference were observed in the groups with soft tissue damage, particularly at the popliteal level and the proximal thigh. Likewise, limb volume in groups 2 and 3 increased progressively over time.

The moderate volume increase observed in both limbs across all groups is likely attributable to the natural growth of the young animals. In the soft tissue injury groups, however, a significant increase in volume and circumference of the operated limbs became apparent only after 14–21 days. This delayed onset likely reflects the gradual accumulation of lymphatic fluid. A similar temporal delay between trauma and symptom onset is frequently observed in patients with lymphedema following tumor resection or lymph node dissection.

The extent to which lymphedema is accompanied by structural tissue changes and the cellular or molecular mechanisms involved should be addressed in future studies using this model. Although translational limitations exist – as findings from animal models cannot be directly extrapolated to humans – this model provides a valuable platform for investigating the effects of lymphedema on bone regeneration.

## 5. Limitations

This study has several limitations that should be acknowledged. First, the translational applicability of the rat model to human posttraumatic lymphedema is inherently limited due to species-specific differences in anatomy and immune response. Second, the current analysis primarily relies on morphometric data; histopathological and functional assessments were not included at this stage. These will be the subject of future studies using this model to investigate underlying pathophysiological mechanisms in more detail. Third, variability in surgical technique may influence the consistency of lymphedema induction, despite standardized protocols. Lastly, the absence of long-term follow-up limits the ability to assess the development and progression of chronic lymphedema.

## 6. Conclusions

To date, no animal model has been established that allows for targeted investigation of the pathophysiology of posttraumatic lymphedema. Moreover, the impact of lymphedema on bone healing remains largely unexplored. The aim of this study was therefore to develop a combined model that reflects the pathophysiological characteristics of trauma-induced lymphedema while enabling simultaneous assessment of bone regeneration.

Approximately 3–4 weeks after surgical disruption of lymphatic vessels and soft tissue trauma, a significant increase in limb circumference and volume was observed, consistent with posttraumatic lymphedema. Microscopic evaluation confirmed that this swelling was due to impaired lymphatic drainage. The presence of a concurrent bone defect did not influence the degree of swelling or volume increase.

This animal model therefore offers a promising platform for future studies investigating the mechanisms underlying trauma-related lymphedema and its effect on bone healing. It may also serve as a basis for evaluating novel therapeutic and regenerative strategies in a clinically relevant context.

## Supporting information

S1 Data Data template. (PDF)
