# Supplementary material for: Establishing a new rat model to investigate pathophysiology and bone healing in posttraumatic lymphedema
Source: PLoS One. 2025 Sep 16;20(9):e0332067. doi: 10.1371/journal.pone.0332067 (PMC12440177; doi:10.1371/journal.pone.0332067)
Supplement: S1 Data — (PDF) [file pone.0332067.s001.pdf]

Data Template

Animal ID	Group	Day 0 right	left	Day7 right	left	Day 14 right	left	Day 21 right	left	Day 28 right	left
		Diameter popliteal (mm)									
**1**	Bone	15,2	11,5	14,6	13,5	15,2	15,2	15,2	14,2	15	16,1
**2**	Bone	13	12,2	15,1	14,2	16	15,5	16,3	17,5	16,4	17,5
**3**	Bone	17	14,8	15,5	15,6	18,9	16,3	19,4	18,6	18,7	19,8
**4**	Bone	11,5	15,5	16,2	19	17,5	17,8	17,8	19,2	19,4	18,6
**5**	Bone	14	16,7	16,4	18,5	16,3	18,7	18,9	17,9	17,8	17,2
**6**	Bone	12,8	13	16,7	17,3	19	16,1	17,1	18,7	17,3	20
**7**	Bone	14,5	12,5	17	16,9	17,8	15,9	20,1	16,4	16,9	18,9
**8**	Bone	15,5	14	17,8	17,1	16,7	17,2	16,7	19,6	18,1	17,7
**9**	Bone	12	15,3	18	15,8	16,1	16,5	17,5	17,3	17,6	18,4
**10**	Bone	16,5	13,5	16	16,1	17,5	17,8	17	18,6	17,8	16,7
**11**	Lymph	16,5	11,7	16	15,6	18,2	15,2	21,2	15,2	20	15,1
**12**	Lymph	11,5	13	15,5	16,2	19,6	17,1	25,8	18,5	22,1	17
**13**	Lymph	15	16,8	18,5	17,8	21,5	19,3	26,4	19,3	24,7	19,4
**14**	Lymph	14,8	14,2	19	18,9	24,8	21	24,3	20,1	25,1	18,6
**15**	Lymph	12,5	15,6	18,3	17	22,1	18,9	24,8	17,4	23,5	17,5
**16**	Lymph	13	13,3	17,8	16,5	23,7	18,5	23,5	18,7	24	16,3
**17**	Lymph	14	14,9	18,9	18,3	25	19,6	26,6	19,9	22,8	20
**18**	Lymph	13,7	15,1	19	17,4	20,3	20,1	24,1	16,8	23,3	17,9
**19**	Lymph	16	12,5	17,6	16,8	23,9	17,8	23,7	18	24,6	16,7
**20**	Lymph	13,1	14,9	17,3	17,5	21,1	18,5	24,6	17,3	21,1	18,5
**21**	Lymph+Bone	16,7	11,9	15,8	15,5	18,2	15,2	22,2	15,2	22	16,1
**22**	Lymph+Bone	12,3	14,8	16,2	16,1	21,5	17,3	24,1	16,1	24,8	17,5
**23**	Lymph+Bone	15,6	16,5	19,6	18,7	25	18	25,6	18,3	25,5	19,2

**24**	Lymph+Bone	14,2	16,9	17,9	17,8	23,9	20,5	27,6	19,7	24,1	20,6
**25**	Lymph+Bone	11,5	13,7	18,7	16,5	24,7	19,6	23,9	17,6	23,6	18,4
**26**	Lymph+Bone	13,9	15	17,4	17,1	22,3	18,2	26,5	18,9	24,3	17,8
**27**	Lymph+Bone	16	14,5	16,9	16,3	23,8	17,9	25,2	16,4	24,7	18,9
**28**	Lymph+Bone	14,5	15,8	18,1	18,9	21	19,1	24,8	20,1	22,9	19,5
**29**	Lymph+Bone	13,7	16,2	17,5	17,4	24,1	16,8	27,1	17,2	23,9	18,1
**30**	Lymph+Bone	15,6	12,7	17,9	16,7	22,2	18,4	23	17,5	23,2	17,9

Diameter thigh (mm)

		**Day 0**		**Day7**		**Day 14**		**Day 21**		**Day 28**	
		**right**	**left**	**right**	**left**	**right**	**left**	**right**	**left**	**right**	**left**
**Animal ID**	**Group**										
**1**	Bone	14,1	14,6	15,6	15	16,6	15,4	17,5	16,3	16,1	18,6
**2**	Bone	15,8	18,2	17,4	18,6	17,9	18,7	19,9	20,1	20,5	22,3
**3**	Bone	17,2	16,9	18,9	17,9	19,5	19,5	22,7	22,5	23,4	23,5
**4**	Bone	16,9	17,5	20	16,3	20,2	20,2	20,4	18,7	18,7	20,1
**5**	Bone	16	15,1	19,5	17,1	18,7	18,3	18,7	19,2	19,1	19,7
**6**	Bone	15,1	18,9	17,8	18,4	18,3	17,6	21	23	24	21,8
**7**	Bone	18,4	16	18,3	16,8	19	19	19,2	17,8	17,8	20,5
**8**	Bone	16,5	15,7	16,7	19	17,5	16,9	20,8	20,4	21,2	22,9
**9**	Bone	16,2	17,3	19	17,5	19,2	17,8	18,9	18,9	18,9	19,2
**10**	Bone	16,8	15,8	18,8	15,6	18,6	18,6	19,3	19,5	19,8	20,4
**11**	Lymph	14,3	14,2	16,6	15,3	22,6	18,4	22,5	17,1	22,1	18,6
**12**	Lymph	15	18,3	20,1	18,7	24	22,5	28,7	20,3	27,4	22,5
**13**	Lymph	17,9	17,5	22	17,4	26,5	24,2	26,1	22,8	28,5	23,9
**14**	Lymph	18,4	19,1	19,4	16,1	25,9	21	27,4	19,5	24,7	21,2
**15**	Lymph	15,6	16,4	21,7	19,2	27,2	19,8	29,9	21	25,8	20,8

**16**	Lymph	16,8	15,3	18,6	16,8	23,3	23,1	25,8	20,7	26,1	22,1
**17**	Lymph	16	17,9	19,8	17	24,8	20,9	26,9	22,4	23,3	23
**18**	Lymph	16,5	16,8	20,3	18,1	25,1	22,7	24,5	19,8	27	19,5
**19**	Lymph	17,3	15,6	19,9	15,9	23,7	21,4	27	18,7	24,2	21,7
**20**	Lymph	15,2	17,1	20,5	17,5	24,1	21,2	26,7	20,9	25,9	21,7
**21**	Lymph+Bone	14,7	13,2	17,6	15,6	22,6	18,5	22,5	17,1	22,4	17,6
**22**	Lymph+Bone	15,3	15,1	19,4	18,3	25,3	22,3	29,7	22,4	27,8	22,5
**23**	Lymph+Bone	18,9	16,7	21,2	19,7	26,7	24,1	26,8	23	28,3	23,9
**24**	Lymph+Bone	17,4	17,9	22	17,1	27,2	23,7	27,9	20,8	24,5	21,7
**25**	Lymph+Bone	16,8	15,4	18,7	20,2	24,9	21,9	30,5	21,7	26,9	20,8
**26**	Lymph+Bone	15,1	18	20,1	18,8	23,8	22,8	25,6	22,9	25,4	22,1
**27**	Lymph+Bone	17,9	14,8	19,9	16,4	25,7	19,5	27,3	21,1	23,7	23
**28**	Lymph+Bone	16,2	16,5	19	19,5	24,1	23,3	24,8	20,9	27,1	19,5
**29**	Lymph+Bone	16,5	15,6	20,3	17,9	25	20,9	28,4	22,3	25,2	21,7
**30**	Lymph+Bone	15,2	16,7	17,3	18,5	24,4	21,4	26,4	21,1	27,7	21,7

Circumference popliteal (cm)

Animal ID	Group	Day 0	Day 7	Day 14	Day 21	Day 28
**1**	Bone	4,2	4,4	4,5	4,3	4,9
**2**	Bone	3,8	4,4	4,3	4,8	4,2
**3**	Bone	4,4	4,3	4,4	4,9	4,4
**4**	Bone	3,9	4,1	4,6	4,6	4,5
**5**	Bone	4,1	3,4	4,6	4,5	4,6
**6**	Bone	3,7	3,9	4,5	4,9	4,7
**7**	Bone	4	4,2	4,1	4,7	4,8
**8**	Bone	4,3	4,3	4,5	4,6	4,3
**9**	Bone	4,1	4	4,6	4,6	4,5
**10**	Bone	3,4	4,7	4,4	4	4
**11**	Lymph	3,3	4,2	4,8	5	5,1
**12**	Lymph	3,8	4,3	5	5,2	5
**13**	Lymph	4,2	4,4	5,3	5,6	5,3
**14**	Lymph	4,4	4,5	5,1	5,4	5,7
**15**	Lymph	4,1	4,7	5,4	5,7	5,9
**16**	Lymph	4	4,8	5,6	5,9	5,8
**17**	Lymph	4,3	4,9	4,9	5,3	5,4
**18**	Lymph	3,7	4,6	5,2	5,5	5,5
**19**	Lymph	4,1	4,5	5,1	5,8	5,2
**20**	Lymph	4,2	4,5	5	5,4	5,6
**21**	Lymph+Bone	3,4	3,9	4,6	5,1	4,7
**22**	Lymph+Bone	3,9	4,1	5	5,3	5,1
**23**	Lymph+Bone	4,3	4,8	5,3	5,7	5,4
**24**	Lymph+Bone	3,7	4,5	5,5	5,9	5

**25**	Lymph+Bone	4	4,7	5,1	5,6	5,3
**26**	Lymph+Bone	3,8	4,2	4,9	5,4	5,2
**27**	Lymph+Bone	4,1	4,4	5,4	5,8	5,4
**28**	Lymph+Bone	3,5	4,6	5,2	5,5	5
**29**	Lymph+Bone	3,8	4,3	5,3	5,5	5,2
**30**	Lymph+Bone	4,1	4,7	5,1	5,3	5,2

		Circumference thigh (cm)				
**Animal ID**	**Group**	**Day 0**	**Day 7**	**Day 14**	**Day 21**	**Day 28**
**1**	Bone	4,9	5,3	5,6	4,5	4,7
**2**	Bone	4,2	5,1	4,5	5,6	5,3
**3**	Bone	4,5	5,3	5	5	5,4
**4**	Bone	4,7	5	5,2	5,2	5,6
**5**	Bone	4,3	5,6	5,3	5,4	5,2
**6**	Bone	4,1	4,8	4,7	4,9	5,5
**7**	Bone	4,6	5,2	5,1	5,3	5,1
**8**	Bone	4	4,5	4,9	5,1	5,4
**9**	Bone	4,4	4,9	5,4	5,2	5,6
**10**	Bone	4,7	5,1	4,8	5,4	5
**11**	Lymph	3,5	4,5	4,9	5,5	5,5
**12**	Lymph	3,9	5	5,3	6,1	5,8
**13**	Lymph	4,3	5,4	5,7	6,3	6,1
**14**	Lymph	4,6	4,9	5,9	6,7	6,3

**15**	Lymph	4,2	4,7	5,5	6,4	6
**16**	Lymph	3,8	5,1	5,2	6,2	5,9
**17**	Lymph	4,1	5,3	5,8	6	6,7
**18**	Lymph	4	4,8	5,4	6,5	6,2
**19**	Lymph	4,4	5,2	5,5	6,3	6,4
**20**	Lymph	4,1	4,7	5,3	6,2	5,9
**21**	Lymph+Bone	4,1	4,6	4,8	6,3	6,3
**22**	Lymph+Bone	4,2	4,1	5	5,9	5,9
**23**	Lymph+Bone	4,3	4,9	5,1	6,4	6,4
**24**	Lymph+Bone	4,4	4,7	5,2	6,1	6,1
**25**	Lymph+Bone	4,5	4,3	5,3	5,7	5,7
**26**	Lymph+Bone	4,6	5,1	5,4	6	6
**27**	Lymph+Bone	4,7	4,5	5,5	5,8	5,8
**28**	Lymph+Bone	4,8	4,8	5,6	6,1	6,4
**29**	Lymph+Bone	5	4,4	5,7	5,5	5,5
**30**	Lymph+Bone	4,4	4,6	6,4	6,2	6,2

Volume thigh (g)

		Day 0		Day7		Day 14		Day 21		Day 28	
**Animal ID**	**Group**	**right**	**left**	**right**	**left**	**right**	**left**	**right**	**left**	**right**	**left**
**1**	Bone	4,5	4,3	4,3	4,6	4,7	4,5	4,6	4,5	5,4	4,4
**2**	Bone	4,4	4,1	4,1	4,1	4,6	4,1	4,3	4,3	4,1	4,5
**3**	Bone	4,2	4,5	4,6	4,3	5	4,9	4,8	4,9	4,8	4,7
**4**	Bone	4,7	3,9	4,7	3,9	4,2	4,3	4,9	4,4	4,7	5,4

**5**	Bone	4,1	4	4,2	4,2	4,8	5	4,1	4,2	5	4,6
**6**	Bone	4,4	4,4	6,8	4,4	4,4	4,8	4,4	4,8	5,3	4,3
**7**	Bone	4	4,2	4,5	5,3	4,6	4,4	4,5	4,7	4,3	4
**8**	Bone	4,6	4,3	4,4	4	5,1	4,7	4,7	5	4,6	4,1
**9**	Bone	4,3	4,5	4,6	4,7	4,9	4,7	4,2	4,6	4,5	4,5
**10**	Bone	4,7	4,8	4,8	4,5	4,7	4,6	4,5	4,6	5,3	4,5
**11**	Lymph	4,4	3,8	4,5	4,5	5	3,8	5,6	4,2	5,5	4
**12**	Lymph	4	4,4	5	4	5,3	4	5,8	3,9	5,1	4
**13**	Lymph	4,9	4,5	4,2	4,6	5,5	4,1	5,2	4,1	4,6	4,1
**14**	Lymph	4,3	4,2	4,9	4,9	5,2	4,2	5,4	4,3	5,2	4,1
**15**	Lymph	4,3	4,3	4,7	4,1	5,6	4,2	4,8	3,8	5,4	4,2
**16**	Lymph	4	4,4	4,6	3,9	5,4	4,3	5,5	4,4	4,8	4,2
**17**	Lymph	4,6	3,9	4,8	4,4	5,6	4,3	5,9	4,6	5,3	4,3
**18**	Lymph	4,7	4	4,8	4,6	5,5	4,4	5	4,5	5	4,3
**19**	Lymph	4,5	4,1	5,1	4,2	6,4	4,4	5,7	4	5,7	4,4
**20**	Lymph	4,2	5,9	4,4	4,8	5,3	4,5	5,9	4,2	5,4	4,4
**21**	Lymph+Bone	4,9	4,3	5,3	4,5	5,1	4,4	5,2	4,2	5,2	4,9
**22**	Lymph+Bone	4,1	3,8	4,7	4,7	5,6	3,9	5,6	4	5,6	4
**23**	Lymph+Bone	4	4,2	4,8	4,1	5,3	4,2	5,4	4,1	5,4	4,4
**24**	Lymph+Bone	4,7	4,1	5	4,3	5	4,6	4,9	4,5	4,9	4,3
**25**	Lymph+Bone	5,3	4	4,9	4,2	5,7	4,3	5	4,3	5	5,3
**26**	Lymph+Bone	3,9	4,2	4,9	4,8	4,9	4,6	5,3	4,4	5,3	4,8
**27**	Lymph+Bone	4,9	4,5	4,2	3,9	5,4	4,5	5,1	4,6	5,1	4,1
**28**	Lymph+Bone	5	4,6	5,1	4,6	5,5	4	5,5	5,4	5,5	5
**29**	Lymph+Bone	4,4	3,9	4,9	4,4	5,3	4,4	4,8	4	4,8	4,5
**30**	Lymph+Bone	4,8	4,4	5,2	4,5	5,2	4,5	5,3	4,5	5,3	4,7
